# Short-Lived Human Umbilical Cord-Blood-Derived Neural Stem Cells Influence the Endogenous Secretome and Increase the Number of Endogenous Neural Progenitors in a Rat Model of Lacunar Stroke

**DOI:** 10.1007/s12035-015-9530-6

**Published:** 2015-11-25

**Authors:** Anna Jablonska, Katarzyna Drela, Luiza Wojcik-Stanaszek, Miroslaw Janowski, Teresa Zalewska, Barbara Lukomska

**Affiliations:** 1 NeuroRepair Department, Mossakowski Medical Research Centre, Polish Academy of Sciences, Warsaw, Poland; 2 Department of Radiology and Radiological Science, School of Medicine, Johns Hopkins University, Baltimore, MD USA; 3 Cellular Imaging Section, Institute for Cell Engineering, Johns Hopkins University, Baltimore, MD USA

**Keywords:** Stem cells, Rat model, Lacunar stroke, Growth factors

## Abstract

Stroke is the leading cause of severe disability, and lacunar stroke is related to cognitive decline and hemiparesis. There is no effective treatment for the majority of patients with stroke. Thus, stem cell-based regenerative medicine has drawn a growing body of attention due to the capabilities for trophic factor expression and neurogenesis enhancement. Moreover, it was shown in an experimental autoimmune encephalomyelitis (EAE) model that even short-lived stem cells can be therapeutic, and we have previously observed that phenomenon indirectly. Here, in a rat model of lacunar stroke, we investigated the molecular mechanisms underlying the positive therapeutic effects of short-lived human umbilical cord-blood-derived neural stem cells (HUCB-NSCs) through the distinct measurement of exogenous human and endogenous rat trophic factors. We have also evaluated neurogenesis and metalloproteinase activity as cellular components of therapeutic activity. As expected, we observed an increased proliferation and migration of progenitors, as well as metalloproteinase activity up to 14 days post transplantation. These changes were most prominent at the 7-day time point when we observed 30 % increases in the number of bromodeoxyuridine (BrdU)-positive cells in HUCB-NSC transplanted animals. The expression of human trophic factors was present until 7 days post transplantation, which correlated well with the survival of the human graft. For these 7 days, the level of messenger RNA (mRNA) in the analyzed trophic factors was from 300-fold for CNTF to 10,000-fold for IGF, much higher compared to constitutive expression in HUCB-NSCs in vitro. What is interesting is that there was no increase in the expression of rat trophic factors during the human graft survival, compared to that in non-transplanted animals. However, there was a prolongation of a period of increased trophic expression until 14 days post transplantation, while, in non-transplanted animals, there was a significant drop in rat trophic expression at that time point. We conclude that the positive therapeutic effect of short-lived stem cells may be related to the net increase in the amount of trophic factors (rat + human) until graft death and to the prolonged increase in rat trophic factor expression subsequently.

## Introduction

Stroke poses a major clinical problem. There are several types of strokes, and, with regard to the severity of tissue damage, there is a continuum from a malignant to a lacunar stroke. Lacunar stroke is caused by the occlusion of single penetrating small arteries [1] and, very often, is associated with cognitive and functional impairment [2–5]. While lacunar stroke constitutes a significant portion of all strokes (over 25 %), it has not been well studied. This is, in part, related to the paucity of small animal models. Thus, our group developed a reproducible, reliable, and efficient method of ischemia-like injury in the deep-brain structures of rodents that closely mimics the lacunar stroke [6]. In the last few years, this model has been successfully used by other groups to study the safety and functionality of MSC transplantation [7, 8].

There are virtually no treatment options for patients with lacunar strokes; thus, attempts to restore damaged tissue have been proposed. There is growing evidence that stem cell-based therapy can be a viable option for stroke survivors [9–11]. It has been shown that transplanted stem cells can survive and increase neurogenesis in animal models of stroke [12]. However, multiple reports, also from our group, show difficulties in maintaining long-term graft survival in adult animals [13–17], despite the heavy immunosuppression regime [18]. Yet, despite the absence of transplanted human cells, we and other authors observed positive behavioral effects on a rat model of stroke [19–21]. A similar phenomenon has been observed in an experimental autoimmune encephalomyelitis (EAE) model by two separate groups of investigators [22, 23], where short-lived glial-restricted precursors were shown to have immunomodulatory abilities. Despite the lack of transplanted cell survival, positive effects from umbilical cord-blood-isolated stem cell transplantation were described in an ALS model [24], acute spinal cord injury [25], and brain hypoxia-ischemia [26].

It has been shown that trophic factors, which have neurotrophic, neuroprotective, angiogenic, and anti-apoptotic properties, are important in the regulation of repair processes in pathological conditions. Stimulation of the proliferation of endogenous neural stem cells, as well as the enhancement of the survival of newly generated neuroblasts, accompanied by an increase in the expression of neurotrophic factors, has been earlier described in a rat middle cerebral artery occlusion (MCAO) model [27, 28]. The most commonly evaluated trophic factors in experimental models of ischemia include BDNF, GDNF, EGF, FGF-2, VEGF, and IGF-1 [29, 30].

We have previously shown an increase in neurogenesis in stroke after the intracarotid delivery of human umbilical cord-blood-derived neural stem cells (HUCB-NSCs) [19]. It has been also postulated by other authors that human cord-blood-isolated stem cells can exert neuroprotective effects either through inhibiting apoptosis or through the production of trophic factors in amyloid-β-induced cognitively impaired mice [31]. In the current study, we investigated whether this increase in neurogenesis is also present after intracerebral transplantation of HUCB-NSCs, and whether it is facilitated by the extracellular matrix changes induced by metalloproteinase activity (MMP). Moreover, we focused on the mechanisms that might mediate the phenomenon of persistent positive treatment effects from short-lived stem cells, through a separate measurement of the expression of human and rat trophic factors.

## Materials and Methods

### Animals

The experiments were performed on adult male Wistar rats weighing 250 g. A total of 120 animals were used for all the experiments, with five animals per every analyzed group in a single experiment (Table 1). Throughout the experiments, animals were housed in plastic cages with a 12-h light-dark cycle and free access to food and water. All procedures complied with EU guidelines for the use of animals in research and were approved by the Fourth Warsaw Local Ethics Committee.

**Table 1:**
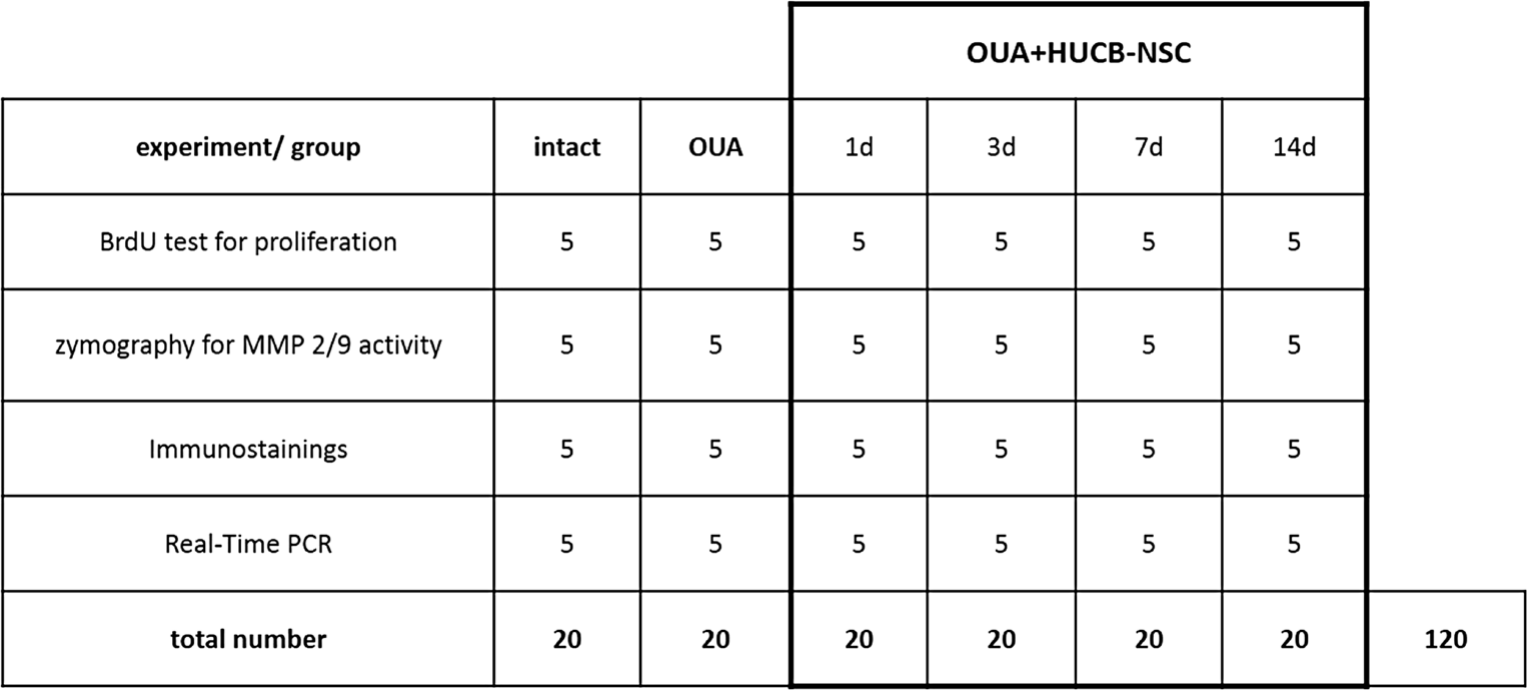
Number of animals used for all experimental groups

### Human Umbilical Cord-Blood-Derived Neural-Like Stem Cell Line (HUCB-NSC) Culture

A neural stem cell line was derived from human umbilical cord blood [32] and cultured in low serum (LS) medium containing Dulbecco’s modified Eagle’s medium (DMEM) and F12 (Gibco) supplemented with 2 % fetal bovine serum (FBS; Gibco), insulin, transferrin, and selenium (ITS, 1:100; Gibco), and an antibiotic antimycotic solution (AAS, 1:100; Sigma). Under these conditions, HUCB-NSCs grew as two subpopulations, floating and adherent cells. At 24 h before transplantation, HUCB-NSCs were moved into a serum-free Neurobasal (NB) medium with B27 to reduce serum levels and the risk of a recipient immunological reaction. Only floating HUCB-NSCs were taken for transplantation.

### HUCB-NSCs Labeling with a CMFDA Cell Tracker

HUCB-NSCs preconditioned with NB medium and B27 for 24 h were collected and suspended in NB fresh medium, and 10 mM 5-chloromethyl-fluorescein-diacetate (CMFDA; 1 μl/500 μl NB) cell tracker was added. The cells were incubated at 37 °C for 30 min, washed twice with serum-free NB medium, and counted using a light microscope. Immediately before transplantation, HUCB-NSCs were re-suspended in saline and assessed with the trypan blue exclusion test for viability, which was not lower than 95–98 % in all experiments.

### Rat Model of Lacunar Stroke

Rats were anesthetized with ketamine (90 mg/kg) and xylazine (10 mg/kg), given by i.p. injection, and immobilized in a stereotaxic apparatus (Stoelting). A small burr hole was drilled in the cranium over the right hemisphere. The needle (length 15 mm, gage 33), connected to a 10-μl syringe (Hamilton, Switzerland), was lowered into the right striatum (coordinates A 0.5, L 3.8, D 4.7 mm). To minimize brain shift, there was a delay of 5 min between the needle insertion and the injection of the active substance. Then, 1 μl of 5 nmol ouabain (Sigma, Poland) was injected into the brain at a rate of 0.5 μl/min using a microinfusion pump (Stoelting, USA) mounted on a stereotaxic apparatus (Stoelting), as previously described [6]. The needle was then withdrawn and the skin was closed with a suture.

### HUCB-NSC Transplantation

Three days after ouabain injection, rats were anesthetized with ketamine (90 mg/kg) and xylazine (10 mg/kg), given by i.p. injection, and immobilized in a stereotactic apparatus (Stoelting). Then, an incision was made through the skin overlying the sagittal suture, and a small burr hole was drilled in the cranium over the right hemisphere. The needle (length 15 mm, gage 33), connected to a 5-μl Hamilton syringe, was lowered into the corpus callosum (coordinates A 0.0, L 4.0, V 3.0 mm where the bregma was adjusted to the same horizontal plane, and the ventral coordinates were calculated from the dura), and 2 μl of HUCB-NSCs labeled with CMFDA (2 × 10^5^) was injected at a rate of 0.5 μl/min via a microinfusion pump (Stoelting) mounted on stereotactic apparatus. After injection, the needle was left in situ for 5 min to avoid the leakage of injected cells through the needle tract. Then, the needle was withdrawn and the skin closed with a suture.

### qRT-PCR Analysis

Total RNA from 1 × 10^5^ HUCB-NSCs or rat brain tissue was extracted using TRIzol (Invitrogen) reagent; contaminating genomic DNA was further eliminated by DNase (Qiagen) digestion according to the manufacturer’s instructions. This RNA (1 μg) sample was used for complementary DNA (cDNA) preparation, using a High Capacity RNA-to-cDNA kit (Applied Biosystems). RT-PCR reactions were carried out using template cDNA in the presence of specific rat or human primers for neuromorphogens and trophic factors. To analyze messenger RNA (mRNA) level, real-time PCR was performed with the 7500 Real-Time PCR system (Applied Biosystems). As a reference gene, the glyceraldehyde-3-phosphate dehydrogenase housekeeping gene (GAPDH) was used. Results are shown as a relative quantification, which determines the changes in steady-state mRNA levels of a gene across multiple samples and expresses it relative to the levels of an internal control RNA.

$$ R={2^{\hbox{-}}}^{\left[\kern0.1em \varDelta \mathrm{C}\mathrm{T}\kern0.28em \mathrm{sample}\hbox{-} \varDelta \mathrm{C}\mathrm{T}\kern0.28em \mathrm{control}\right]} $$

### Bromodeoxyuridine Injection

Endogenous cell proliferation was determined by bromodeoxyuridine (BrdU) cell-incorporation administered i.p. (50 mg/kg, Sigma-Aldrich), 24 h before rats were anesthetized terminally. 5-Bromo-2-deoxyuridine (BrdU; Sigma-Aldrich) dissolved in physiological saline was administered i.p. (50 mg/kg per injection, in sterile 0.9 % NaCl plus 0.007 N NaOH). Animals received a single dose of BrdU and were sacrificed 24 h after the injection. This procedure was used to determine the number of cells that incorporated BrdU during a 24-h period at a specific time point after ischemia. For BrdU immunostaining, DNA was first denaturated in 2 N hydrochloric acid at 37 °C for 60 min. Then, tissue sections were incubated in 0.1 M sodium tetraborate (pH 8.5) for 15 min, blocked with 10 % normal goat serum in PBS containing 0.25 % Triton X-100 for 60 min, and incubated with anti-BrdU overnight at 4 °C. Following the washing procedure, the primary antibodies were revealed by appropriate secondary anti-rat IgG2a FITC-conjugated antibodies for 60 min at RT and in the dark.

### Brain Tissue Preparation and Fixation

Rats were deeply anesthetized with ketamine (90 mg/kg) and xylazine (10 mg/kg), administered i.p., at 1, 3, 7, and 14 days after HUCB-NSC transplantation. The brains were removed, immediately frozen with dry ice, and stored at −70 °C. Before sectioning, the brains were kept at −20 °C overnight. Coronal tissue sections, 20-μm thick, were cut in a cryostat and mounted on super-frost microscope slides, and then stored at −70 °C until immunohistochemistry was performed. For the immunohistochemical evaluation and in situ zymography, the subventricular zone (SVZ) and subgranular zone (SGZ) regions were analyzed. For gene expression analysis, the brain tissue samples, with a diameter of 3 mm that contained a fragment of the damaged striatum and white matter with transplanted cells, were collected with a biopsy instrument.

### Immunohistochemistry and Confocal Microscopy Analysis

For immunofluorescence analysis, rat brain sections were air-dried at room temperature for 30 min and fixed with freshly prepared 4 % PFA in PBS (pH 7.4) for 15 min. Before incubation with primary antibodies, non-specific binding was blocked with normal goat serum or bovine serum albumin (1:10, diluted with 0.1 % Triton X-100) for 60 min. Then, the primary antibodies were applied and the brain slices were incubated overnight at 4 °C. To identify the migrating neuroblasts, anti-DCX (cell signaling, 1:200) was used. After rinsing in PBS, the rat brain sections were exposed to goat anti-rabbit (Alexa Fluor 594, red) secondary antibody, for 60 min at RT in the dark. In addition, cell nuclei were stained with 5 μM Hoechst 33258. The adjacent sections were used as negative controls. All procedures for negative controls were processed in the same manner except the primary antibodies were omitted. A confocal laser-scanning microscope (Zeiss LSM 510) was used to obtain detailed images of the positively stained cells. A helium–neon laser (543 nm) was used for excitation of Alexa Fluor 594. An argon laser (488 nm) was used for the excitation of CMFDA, and diode 405 nm was used for the excitation of Hoechst. Following acquisition, images were processed using the software package ZEN 2008.

The contribution of specific populations in the rat brain was evaluated using a single, specific antibody stain. The tissue brain sections were analyzed with a confocal microscope and photographed, and then, the 12 photos that were taken at the same magnification were reviewed, and the positive cells were counted. At the same time, all the brain cell nuclei were stained with Hoechst and also counted. These results are shown in the graphs as a percentage of a particular phenotype of cells relative to all the cells of the tissue.

### In Situ Zymography

In order to localize the activity of metalloproteinases (MMP-2 and MMP-9) belonging to the gelatinase family in the rat brain, in situ zymography was performed. Thawed, non-fixed coronal brain sections (25-μm thick) were incubated for 3 h at 37 °C in a humid dark chamber in a reaction buffer containing 50 mg/ml of FITC-labeled DQ-gelatin (Invitrogen Molecular Probes, Eugene, OR), which was quenched intramolecularly. Gelatin-FITC cleavage by tissue metalloproteinase releases peptides whose fluorescence is representative of proteolytic activity. The sections were rinsed in PBS and fixed in cold 4 % PFA for 20 min, and then mounted in fluorescent mounting medium (Dako) and observed using fluorescence microscopy. To confirm that the proteolytic activity was attributable to MMPs, some sections in each experiment were incubated in the above conditions with a broad-spectrum inhibitor of metalloproteinase, 1 mM 1,10-O-phenanthroline.

### Analysis of Pixel Intensity

For quantification of changes in the number of DCX-positive cells and MMP 2/9 activity, confocal pictures at the same FOV size, as well as the same laser intensity and pinhole set up, were made. Every picture was analyzed by measurement of mean pixel intensity, using ImageJ software. Results are presented as an average of five animals’ measurements +/− SD.

### Statistical Analysis

The results are presented as mean ± SD. The number of repetitions is indicated in the descriptions of the figures. In order to test the statistical significance of differences between mean values, the one-way ANOVA and the Bonferroni test were performed. All calculations used the program Prism 3.0. A significance level of less than 0.05 was considered statistically significant.

## Results

### Occurrence of Neurogenesis in the SVZ and SGZ of OUA-Damaged Rat Brain After HUCB-NSC Transplantation

**Fig. 1:**
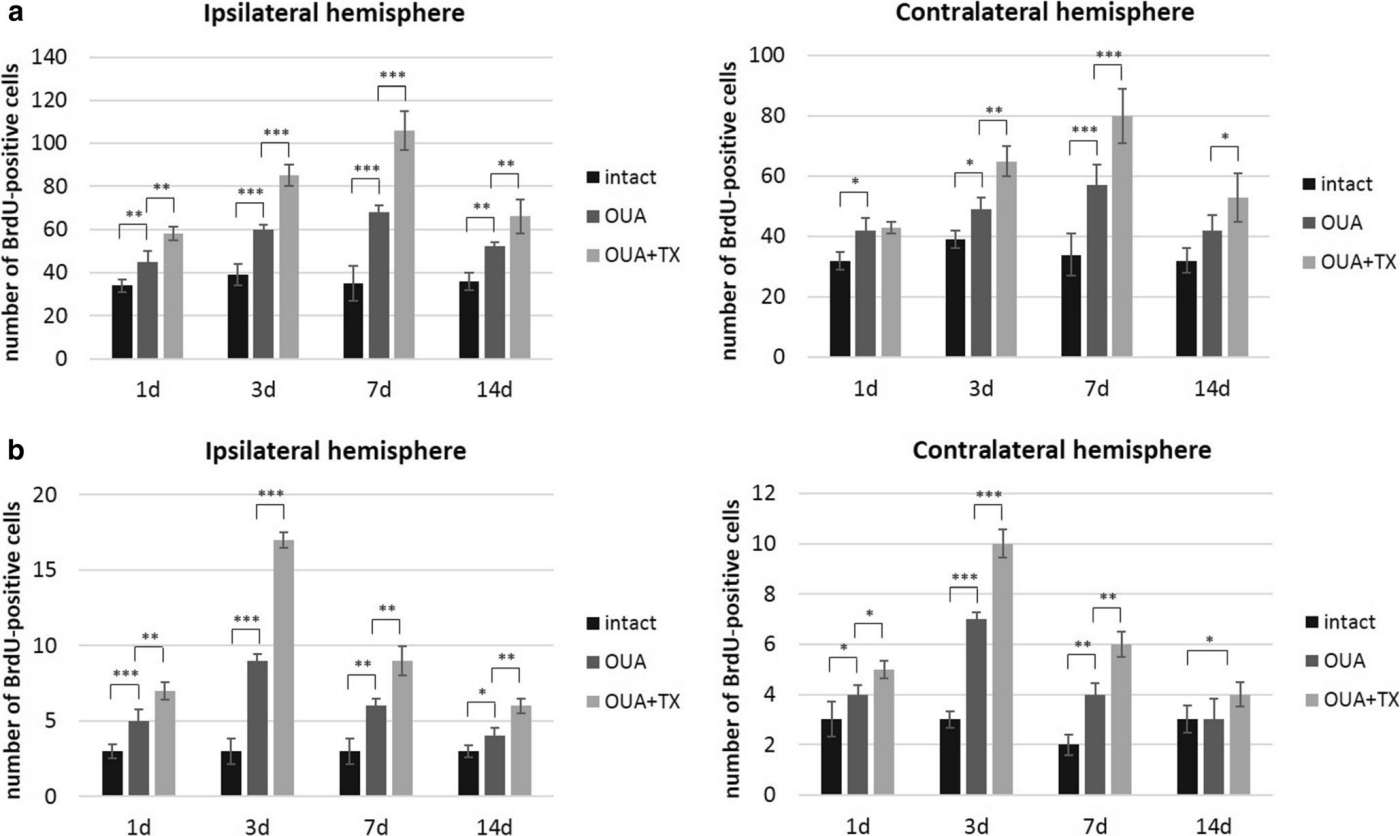
Number of BrdU-positive cells in the SVZ (**a**) and SGZ (**b**) in the rat brain after ouabain injury and HUCB-NSC transplantation. *n* = 5, *p < 0.05; **p < 0.01; ***p < 0.001

OUA-induced brain lesions resulted in increased proliferation (BrdU^+^) and migration (DCX^+^) of newborn cells in the subventricular zone (SVZ) and subgranular zone (SGZ) of the dentate gyrus, compared to intact rats. Transplantation of HUCB-NSCs led to an increase in the number of BrdU^+^ cells compared to ischemic animals. The area covered by BrdU-stained cells delineating the edge of the SVZ and SGZ was markedly augmented the first day after HUCB-NSC transplantation and persisted until the 14th day of the experiment. For the SVZ, at the 1st day after transplantation, the number of BrdU^+^ cells increased from 40 in ouabain-injured animals to 60 per analyzed picture in the ipsilateral hemisphere. The maximum number of proliferating cells was observed at the 7th day, with an increase from 63 in ouabain-injured animals to 107 in animals after transplantation of HUCB-NSC. In the SGZ region, at the 1st day after transplantation, the mean number of cells per analyzed picture was four for injured animals and six in animals after HUCB-NSC transplantation in the ipsilateral hemisphere. In this neurogenic zone, the highest proliferation was observed at day 3 after transplantation, with nine and 16 cells per analyzed picture in ouabain-injured and transplanted animals, respectively. An elevated level of proliferation was observed in both the ipsilateral and contralateral hemispheres; however, the changes in the undamaged side of the brain were about 20 % less pronounced. The increase in the number of proliferating cells was visible in both neurogenic zones, but, in the subventricular zone, it was much higher compared to the area of the SGZ (Fig. 1).

**Fig. 2:**
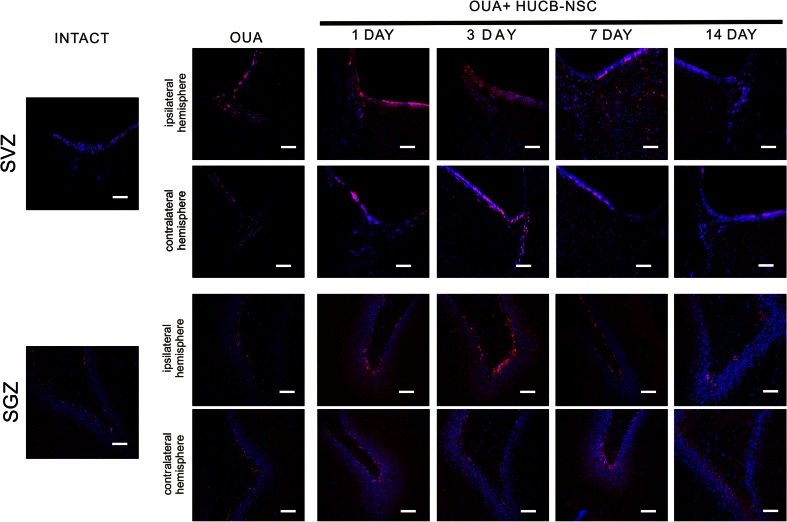
Immunostaining of migrating DCX-positive cells (*red*) in the SVZ and SGZ in the rat brain after ouabain injury and HUCB-NSC transplantation. Nuclei counterstained with Hoechst. *Scale bar indicates* 50 μm. *n* = 5

**Fig. 3:**
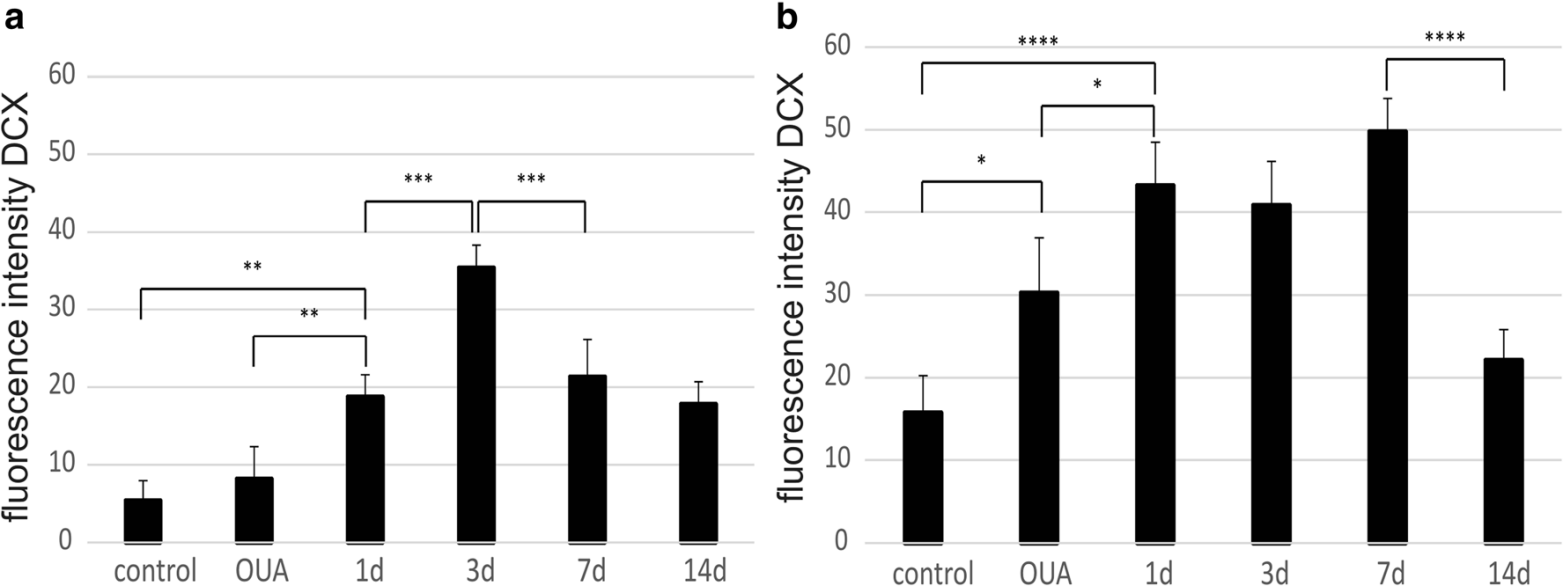
Quantification of pixel intensity representing the number of DCX-positive cells in the SVZ (**a**) and SGZ (**b**')

The increase in the number of BrdU^+^ cells in both the neurogenic analyzed rat brain areas after the administration of ouabain and subsequent HUCB-NSC transplantation was accompanied by an increase in the number of cells showing the presence of a marker characteristic of migratory neuroblasts, doublecortin (DCX). The number of DCX^+^ cells increased significantly in the ipsilateral SVZ and SGZ as rapidly as one day after HUCB-NSC grafting. At the 7th day after HUCB-NSC transplantation, an intense migration of DCX^+^ cells from the SVZ toward the ischemic boundary regions of the striatum was observed. After 14 days, the number of migrating neuroblasts began to drop, which corresponded to a reduced proliferation in the SVZ region on that day (Figs. 2 and 3).

### Activity of Metalloproteinases in the OUA-Damaged Rat Brain After HUCB-NSC Transplantation

**Fig. 4:**
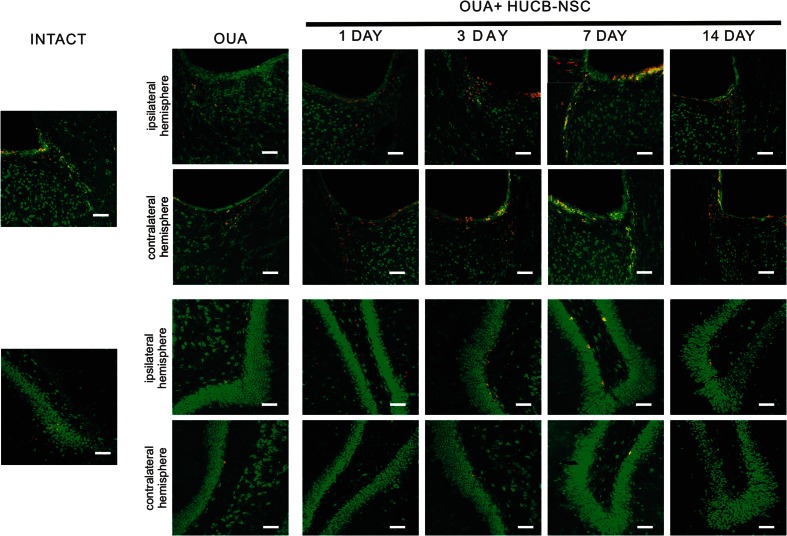
Immunostaining for proliferating BrdU-positive cells (*red*) and in situ zymography for MMP 2/9 activity (*green*) in the SVZ and SGZ in the rat brain in intact animals, as well as after ouabain injury and HUCB-NSC transplantation. *Scale bar* indicates 50 μm. *n* = 5

**Fig. 5:**
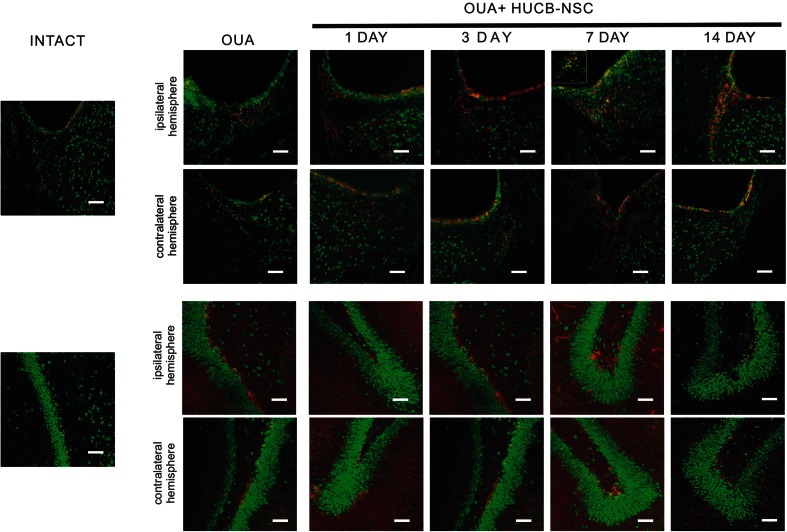
Immunostaining for migrating DCX-positive cells (*red*) and in situ zymography for MMP 2/9 activity (*green*) in the SVZ and SGZ in the rat brain after ouabain injury and HUCB-NSC transplantation. *Scale bar* indicates 50 μm. *n* = 5

**Fig. 6:**
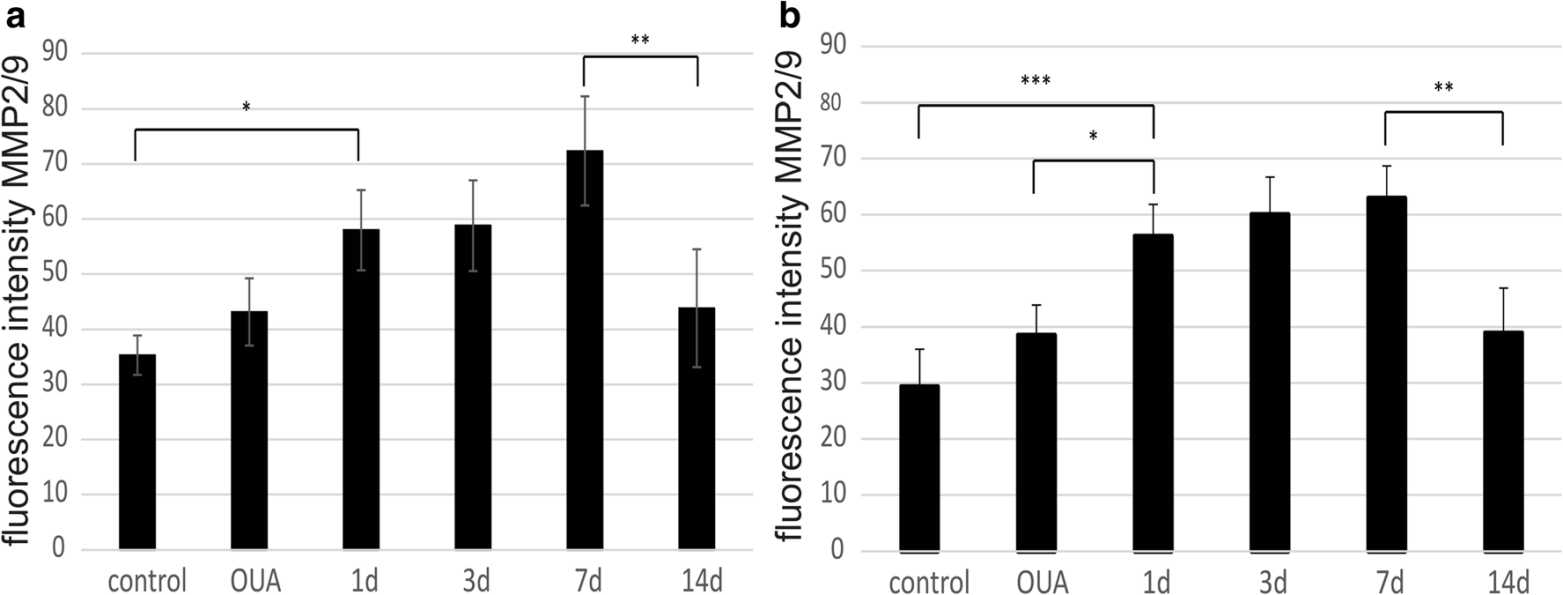
Quantification of pixel intensity representing the activity of MMP 2/9 in the SVZ (**a**) and SGZ (**b**) in the ipsilateral hemisphere

The activity of matrix metalloproteinases 2 and 9 (MMP 2/9) in neurogenic areas of the brain was assessed by in situ zymography. These studies showed an increase in the activity of MMP 2/9 in animals with ouabain striatal damage and in rats after the administration of ouabain and subsequent transplantation of HUCB-NSCs, compared to control animals. The activity of MMP 2/9 in the experimental group of rats with induced striatal damage and transplantation of HUCB-NSCs varied for each day of the experiment. The gradual increase in the activity of MMPs, examined between the 1st and the 7th day after HUCB-NSC transplantation, was particularly apparent in the SVZ area. Over the next few days of observation, the activity of MMP 2/9 decreased and, after 2 weeks, was comparable to that observed in control animals (Figs. 4, 5, and 6).

Double labeling demonstrated the co-localization of MMPs with BrdU^+^ or DCX^+^ cells observed in the SVZ and SGZ. The proteolytic activity of MMPs observed in newborn cells in the SVZ appeared to be associated with the cell nuclei and cytoplasm; however, the presence of MMPs in BrdU^+^ or DCX^+^ cells found in the SGZ was restricted to only the nuclei. High MMP activity was clearly marked in neuroblasts migrating from the SVZ (DCX^+^) within the rostral migration stream (RMS) into the olfactory bulb, and also in cells migrating in the direction of damaged tissue. In the migrating cells, the high activity of MMP 2/9 was visible in the cytoplasm and cell protrusions. In addition, metalloproteinase activity was observed in the extracellular space around the DCX-positive cells, which is likely involved in the loosening of the extracellular matrix that helps cells to migrate through the brain parenchyma (Figs. 4 and 5).

### Lacunar Stroke-Induced mRNA Expression of Endogenous Trophic Factors

**Fig. 7:**
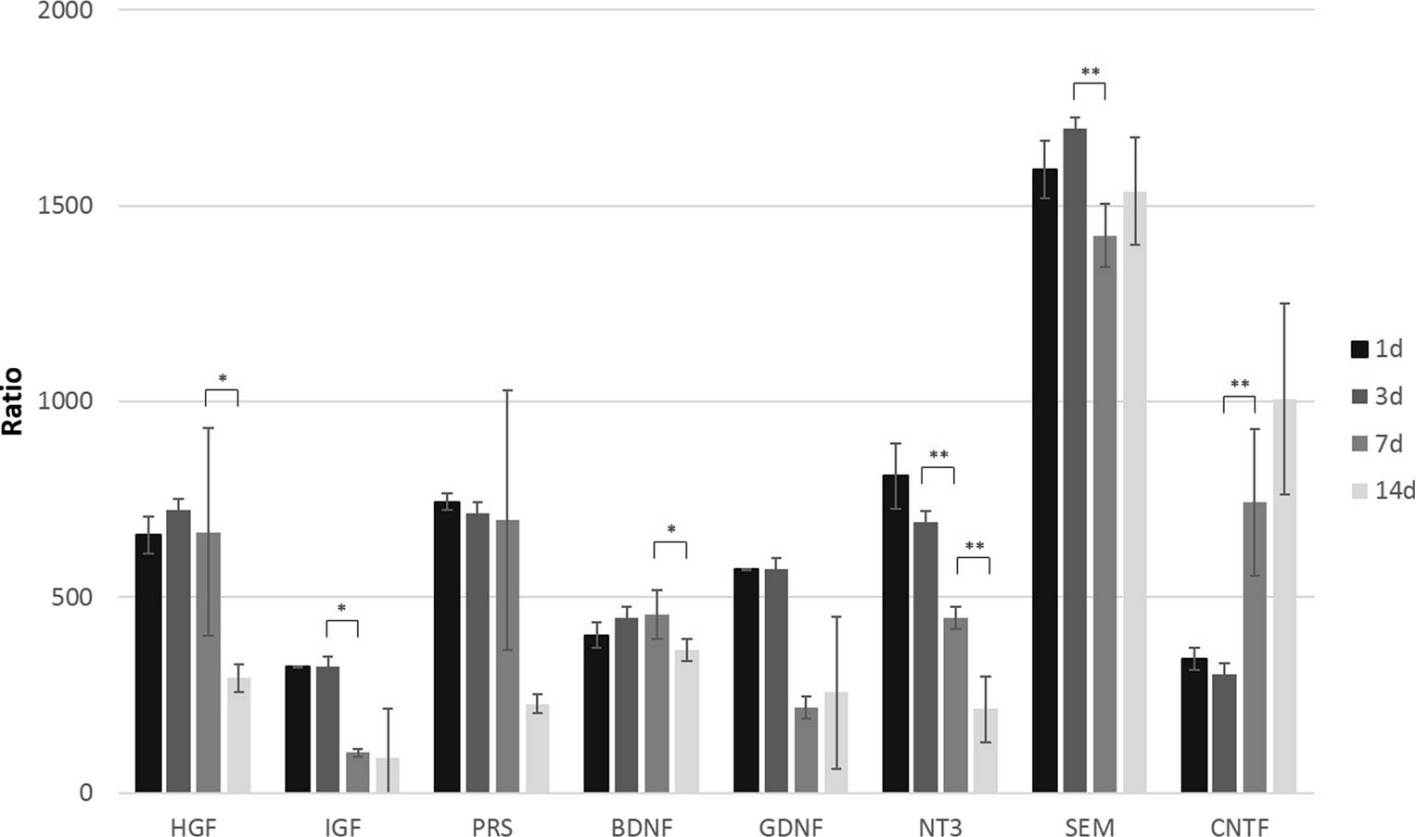
Real-time RT-PCR relative expression of rat trophic factors in ouabain-injured rat brains compared to intact animals. *n* = 5, *p < 0.05; **p < 0.01; ***p < 0.001

We first determined the expression of different trophic factors in the normal and ischemic rat brain. To explore the changes in gene expression, the real-time reverse transcription-PCR (qRT-PCR) method was used to detect mRNA levels of trophic factors (BDNF, GDNF, NT-3, CNTF, SEM, IGF-1, HGF, PRS). As shown in Fig. 7, the administration of ouabain significantly upregulated the endogenous factors in the lesion area, 24 h after brain injury. The calculated ratio of the mRNA level of all factors measured in ischemic and control rat brain exceeded a few hundred-fold. A time course analysis revealed the highest mRNA expression of all molecules except CNTF during the early recovery stage (1–7 days after the insult), which dropped at day 14. The expression of CNTF increased with time after injury and reached the maximum level at the 14th day of the experiment.

### Expression of Human-Origin Trophic Factors in OUA-Damaged Rat Brain After HUCB-NSC Transplantation

**Fig. 8:**
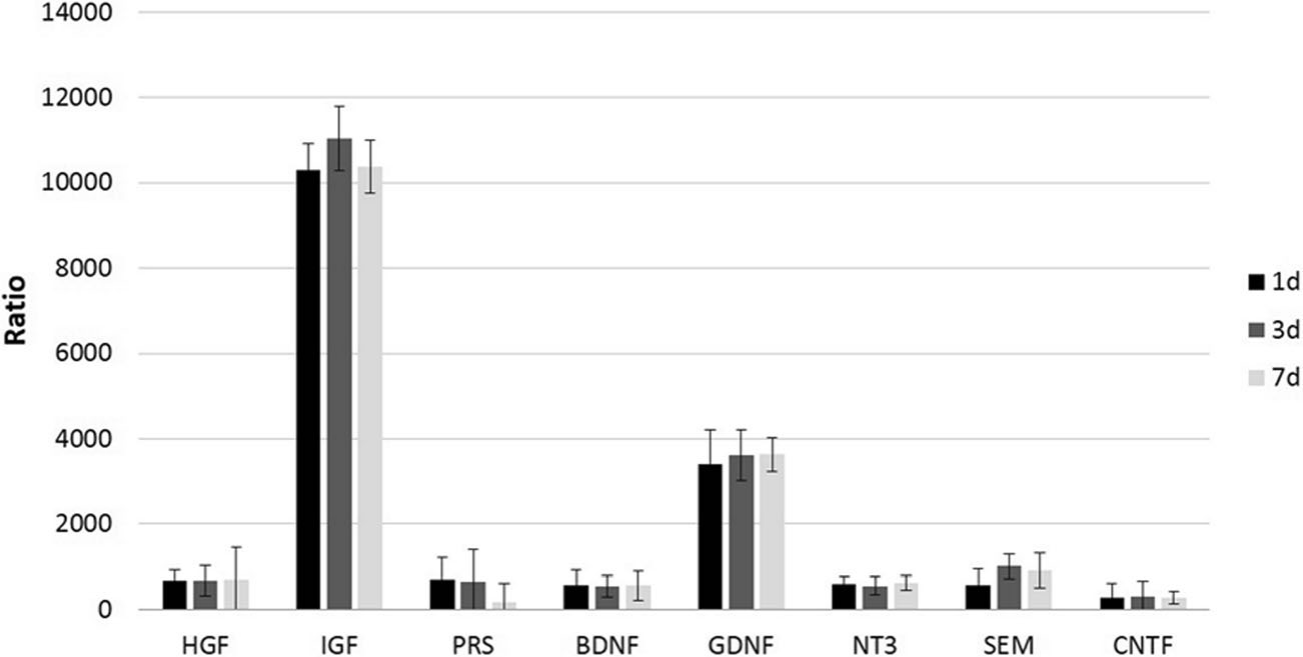
Real-time RT-PCR relative expression of human trophic factors in OUA-injured rat brain after transplantation of HUCB-NSC, compared to constitutive expression by HUCB-NSC in vitro. *n* = 5, *p < 0.05; **p < 0.01; ***p < 0.001

**Fig. 9:**
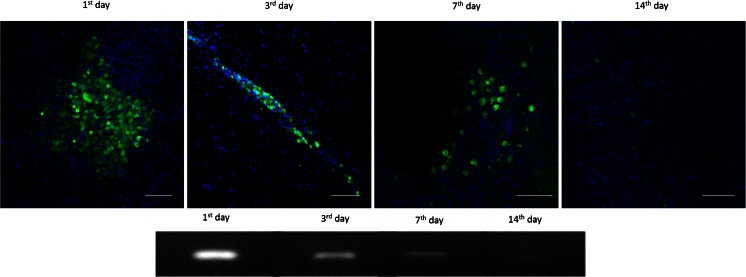
Survival of HUCB-NSC after transplantation into the ouabain-injured rat brain, demonstrated by immunohistochemistry staining (CMFDA cell tracer (*green*), nuclei counterstained with Hoechst (*blue*)) and the presence of human-origin mRNA for GAPDH by RT-PCR

The analysis of the rat brain after ouabain-induced infarction, followed by HUCB-NSC infusion, revealed the presence of trophic factors of human origin. The level of mRNA expression of different factors in the ischemic brain of cell recipients was significantly higher than that of constitutively expressed in HUCB-NSCs cultured in vitro. The calculated ratio of mRNA expression of human-origin trophic factors in the rat brain after HUCB-NSC transplantation to cells themselves in culture, estimated by qRT-PCR, was 300 for CNTF, over 500 for SEM, NT3, BDNF, PRS, and HGF, and the highest increase exceeded 3000 for GDNF and 10,000 for IGF (Fig. 8). The presence of human-origin molecules was observed up to 7 days after the HUCB-NSC graft, probably due to the short time survival of donor cells in the ouabain-damaged rat brain (Fig. 9). There was no significant difference in the mRNA expression of these factors between days 1, 3, and 7 after the implantation of HUCB-NSCs.

### HUCB-NSC Transplantation Prolongs the Increased Expression of Endogenous Trophic Factors Induced by Focal Stroke in the Rat Brain

**Fig. 10:**
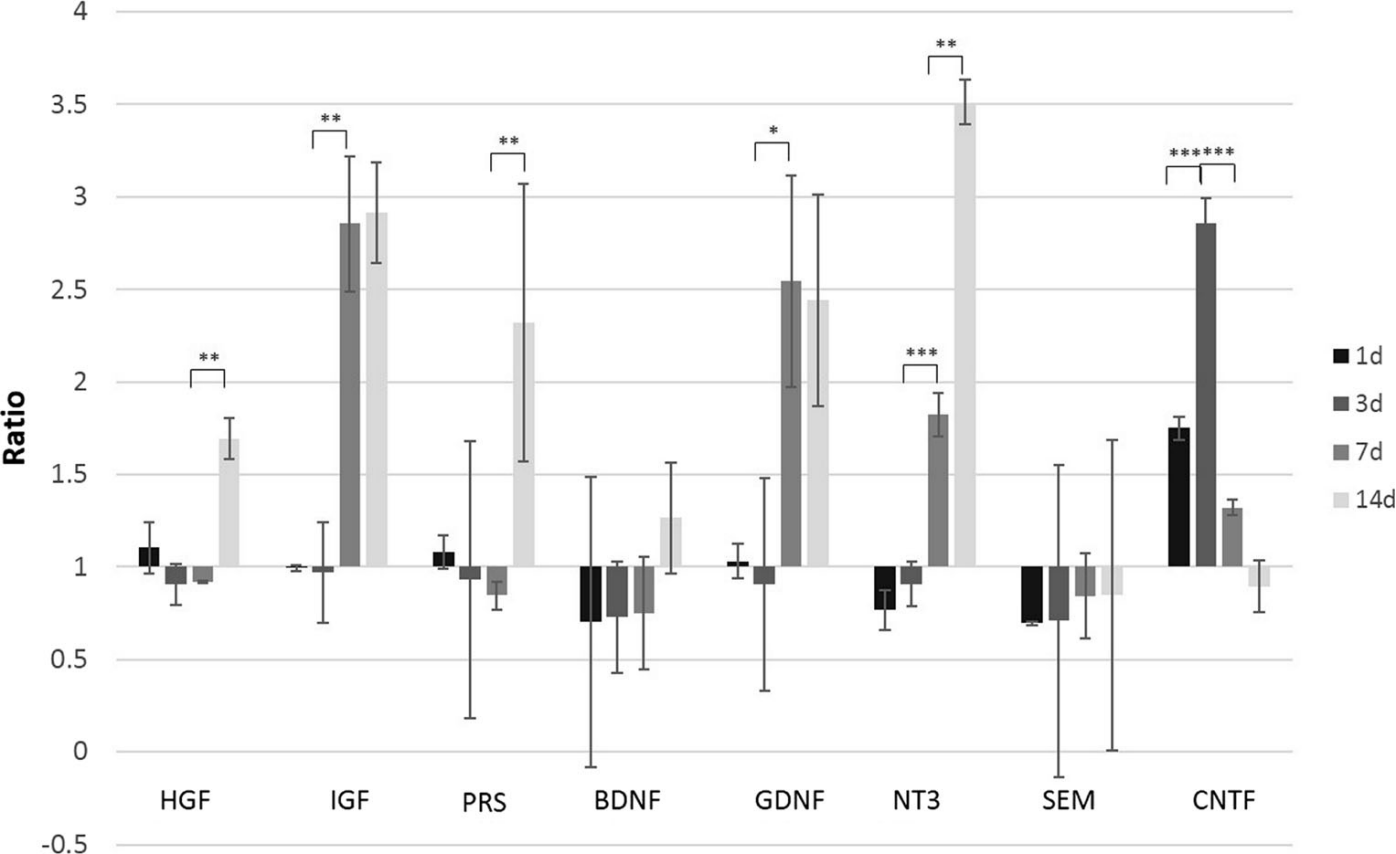
Real-time RT-PCR relative expression of rat-origin trophic factors in ouabain-injured rat brain with HUCB-NSC transplantation compared to ouabain-injured rat brain. *n* = 5, *p < 0.05; **p < 0.01; ***p < 0.001

The transplantation of HUCB-NSCs into the brain of animals with striatal lesions does not change the expression level of rat trophic factors until day 7, compared to ischemic animals without cell implantation. However, the prolongation of increased levels of most rat trophic factors was observed; thus, HUCB-NSCs prevented the drop in trophic factors at a later time point (14 days), except for CNTF (Fig. 10). Only the expression of CNTF was highest at the third day after HUCB-NSC transplantation, and decreased over time. The mRNA level for SEM did not change during the time of observation after HUCB-NSC transplantation, compared to un-transplanted animals.

## Discussion

During the past several years, many studies have shown the positive effects of transplantation of stem cells for the treatment of neurological diseases. The enhancement of endogenous cell proliferation after NSC transplantation has already been demonstrated in a variety of rodent models of brain ischemia [33–36]. In our study, the implantation of HUCB-NSCs into the corpus callosum also increased the number of BrdU-labeled cells in the SVZ and SGZ of rats subjected to a brain insult. In addition, consistent with the proliferation of rat progenitor cells after ischemia, there was migration of newly formed cells to the damaged area. Based on a comparison between the densities of young neuroblasts (DCX^+^) in the rat brain grafted with HUCB-NSCs, the number of newly generated neural precursors was significantly higher after cell transplantation. Among the migrating pool of cells, few co-labeled with the proliferating markers (DCX^+^/BrdU^+^) were found. Similar data has been reported by others [37–39]. With the increase in the number of DCX-positive cells, we also determined whether there were any changes in the migration abilities of newly formed neuroblasts. The mechanism of migration of neural progenitors in pathological conditions of the CNS is not fully understood. There are many factors responsible for this process, including chemoattractant (pro-inflammatory cytokines and chemokines) and matrix metalloproteinases activity (MMPs). The data reported by Barkho et al. (2008) supported the contribution of MMPs to endogenous neurogenesis [40]. The regulatory effect of MMPs on the process of cell migration from the SVZ to the lesion area has been suggested by a number of studies [41–43]. In our experiments, the increased activity of gelatinases (MMP-2 and MMP-9) in cells expressing markers characteristic of young migratory neuroblasts (DCX^+^), compared to the other cells in the brain tissue, seemed to confirm the observations described above. Notably, there was a different localization of MMP-2 and MMP-9 activity between migrating (DCX^+^) and proliferating (BrdU^+^) cells. In our studies, BrdU-positive cells revealed the presence of MMPs only in cell nuclei, which is consistent with these other reports [44–46]. Such localization may indicate that MMPs participate in the regulation of the cell cycle. Our previous results, which showed a decrease in proliferation of the human neural stem cells cultured in vitro in the presence of SB-3CT-mediated inhibition of MMPs activity, support this hypothesis [47, 48]. Unlike BrdU-positive cells, the immune reactivity of MMPs observed in DCX-labeled cells was also confined to the cytoplasm, cell processes, and extracellular space around neuroblasts. The higher activity of MMPs observed in the ischemic rat brain after HUCB-NSC transplantation may facilitate newly formed precursor relocation from neurogenic regions to the site of injury by proteolytic remodeling of the extracellular matrix (ECM). Our immunohistochemical data suggest that MMP-2/9 plays a role in this phenomenon. In vitro studies have shown that the changes in the structure and composition of the ECM affect not only the physical process of cell migration but also signal transmission, through the conversion of inactive forms of trophic factors entrapped in the ECM into their biologically active forms [49, 50].

Although many reports show a positive outcome after stem cell transplantation, the question about the mechanism driving this effect remains unresolved. There is some evidence showing that the presence of donor cells in the brain is not necessary for their positive effect to be exerted on the neurogenesis process. It has been shown that, even after the rapid elimination of transplanted cells [23], or after intravenous transplantation when no donor cells were observed in the host brain [19], the decrease in lesion size and the improvement in behavior were still observed. It seems that transplanted cells work indirectly in the host tissue. Trophic factor secretion is postulated as the primary or supplementary mechanism of action for many transplanted cells; however, there is little direct evidence to support trophin production by transplanted cells in situ. In our studies, the damage of the striatum with ouabain caused an increase in the expression of factors such as BDNF, GDNF, NT-3, CNTF, EGF, HGF, and IGF-1, and in the number of BrdU^+^ cells in the SVZ and the SGZ, compared to the expression in the brain of control animals. Similarly, increased expression has also been observed by other authors. In the studies with animal models of stroke, an increase in the expression of BDNF [51], GDNF [52], HGF, EGF, FGF-2 [53], and IGF-1 [54, 55], as rapidly as 12 h after the insult and lasting until 5 days after stroke, has been described. After transplantation of HUCB-NSCs, the expression of human trophic factors can be detected. The short expression of human trophic factors corresponds well with the short lifespan of HUCB-NSCs previously reported [18]. Interestingly, the human trophic factors only minimally increase the amount of endogenous trophic factors within the first few days, when their expression is high. But, the animals treated with HUCB-NSCs present a prolonged, increased expression of endogenous factors until at least 2 weeks, when untreated animals experience a rapid decrease. These changes in the mRNA levels were also observed by other authors and were independent of the route of cell administration. Increased levels of BDNF, NT-3, and VEGF were found 7–14 days after brain injury and transplantation of the human umbilical cord-blood cells in rodents, but no distinction between human and rat trophic factors was performed in those studies [56, 57]. Borlongan and colleagues have shown that 3 days after intravenous administration of human umbilical cord-blood cells, there was an increased expression of BDNF, GDNF, and NGF in the rat brain after ischemia, compared to control animals. But, later time points were not investigated [58]. Also, intraparenchymal transplantation of human adipose-derived stem cells promotes the expression of the total amount of trophic factors in the rat after cerebral ischemia-reperfusion injury until 28 days after cell delivery [59].

In contrast to the above studies, we compared the time course of expression of human and endogenous factors. In our study, the expression of all analyzed factors, except CNTF, was increased at the 7th or 14th day after transplantation, at a time when, in stroke animals without transplantation, a decrease in the amount of mRNA was observed. This higher and longer expression in the OUA + HUCB-NSC group of animals correlates with a significantly greater number of proliferating cells in the neurogenic zones, compared to OUA-injured rats. A similar effect on proliferation was observed after human bone morrow MSC transplantation into animals after MCAO. Bao and co-workers described an increase in the number of BrdU^+^ cells and in the expression of BDNF, NT3, and VEGF in the animals after MCAO and stem cell transplantation [56]. However, the mechanism of action of exogenous cells after their transplantation into the CNS is not fully understood. As mentioned earlier, there is still not enough evidence for the expression of trophic factors by transplanted human cells in the host tissue. In the literature, there are just a few examples showing that, after transplantation, cells retain their ability to secrete trophic factors. With immunohistochemistry, it has been shown that human MSCs can produce BDNF after their transplantation into a rat model of stroke. Hawryluk and co-workers showed that re-isolated neural precursor cells after transplantation into the damaged spinal cord express a greater amount of several analyzed neurotrophic factors, including CNTF, EGF, and FGF [60]. Wakabayashi et al. also described the expression of human IGF-1 in the rat brain 3 days after MCAO and human MSC intravenous transplantation [61]. In our study, we were able to detect the expression of all analyzed human-origin trophic factors. This expression was observed until the 7th day after transplantation, the day when live donor cells were observed in the rat brain. The level of mRNA for human-origin factors was significantly greater than that in the HUCB-NSC under standard culture conditions, which suggests auto- and paracrine effects of trophic factors on their own expression, or an increase of expression due to conditions of stress—the transplanted dying cells may be subjected to the same mechanisms of trophic factor increase as dying rat cells. The early boost in human trophic factors may prolong the endogenous expression of trophic factors. However, there is also the possibility that other mechanisms, directly related to the death of transplanted cells, such as exosomes [62] or apoptotic bodies, may increase the expression of endogenous trophic factors and provide a positive outcome, as frequently reported in the literature. Actually, the death of transplanted cells may increase the reparative mechanisms, analogously to the endogenous reparative mechanisms that are induced by focal brain injury.

In conclusion, the stimulation of a neurogenic response following a HUCB-NSC graft far exceeds the initial generation of endogenous cells evoked by brain ischemia. The therapeutic benefit of human stem cell transplantation relies on the ability to potentiate neurogenesis and the upregulation of a plethora of trophic factors in graft recipients. It is not known which trophic actions that human factors released by donor cells can reveal to endogenous neural stem/progenitor cells, but we know that they can stimulate higher and longer expression of endogenous trophic factors. The relation of neurogenesis and trophic factor expression is still not clear, since both processes occur at the same time. Thus, further studies are needed to link these processes.
